# Aziridination-Assisted Mass Spectrometry of Nonpolar Sterol Lipids with Isomeric Resolution

**DOI:** 10.1021/jasms.3c00161

**Published:** 2023-07-31

**Authors:** Erin Hirtzel, Madison Edwards, Dallas Freitas, Ziying Liu, Fen Wang, Xin Yan

**Affiliations:** † Department of Chemistry, Texas A&M University, College Station, Texas 77843, United States; ‡ Center for Translational Cancer Research, Texas A&M University, Houston, Texas 77030, United States

## Abstract

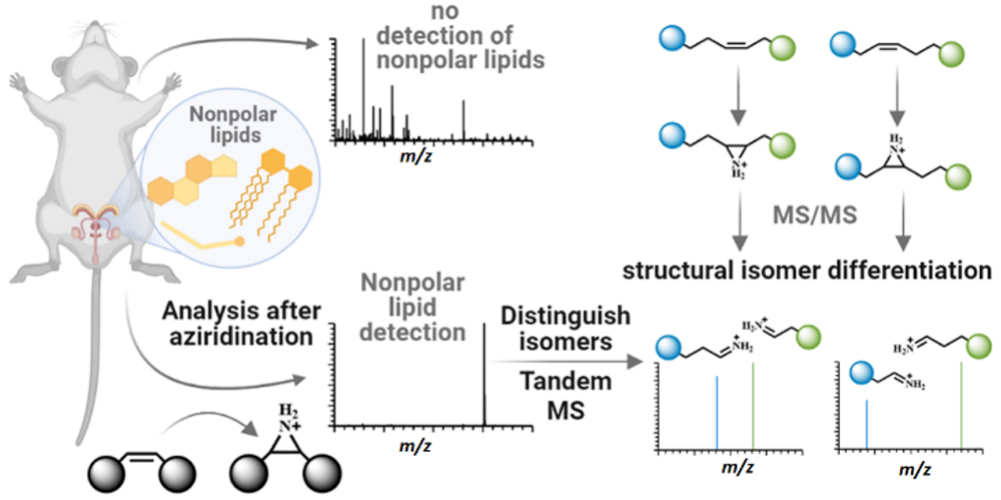

Characterization of nonpolar lipids is crucial due to their essential biological functions and ability to exist in various isomeric forms. In this study, we introduce the N–H aziridination method to target carbon−carbon double bonds (C=C bonds) in nonpolar sterol lipids. The resulting fragments are readily dissociated upon collision-induced dissociation, generating specific fragment ions for C=C bond position determination and fingerprint fragments for backbone characterization. This method significantly enhances lipid ionization efficiency, thereby improving the sensitivity and accuracy of nonpolar lipid analysis. We demonstrated that aziridination of sterols leads to distinctive fragmentation pathways for chain and ring C=C bonds, enabling the identification of sterol isomers such as desmosterol and 7-dehydrocholesterol. Furthermore, aziridination can assist in identifying the sterol backbone by providing fingerprint tandem mass spectra. We also demonstrated the quantitative capacity of this approach with a limit of detection of 10 nM in the solvent mixture of methanol and water. To test the feasibility of this method in complex biological samples, we used mouse prostate cancerous tissues and found significant differences in nonpolar lipid profiles between healthy and cancerous samples. The high efficiency and specificity of aziridination-assisted mass spectrometric analysis, as well as its quantitative analysis ability, make it highly suitable for broad applications in nonpolar lipid research.

## Introduction

Lipids are essential biomolecules that serve a multitude of crucial biological functions, including forming cellular barriers, storing energy, and facilitating intercellular signaling.^1^ Their great diversity of structures and physiochemical properties underpins their various biological roles.^1^ In recent years, nonpolar lipids, such as cholesterol and its derivatives, have gained significant recognition for their involvement in disease detection, progression, and treatment.^2−7^ Cholesterol is essential for membrane biogenesis, and increased cholesterol biosynthesis and uptake have been linked to many types of cancers, therefore becoming a biomarker for cancer progression.^2−11^ Moreover, recent studies have shown that manipulation of cholesterol metabolism could be a promising therapeutic strategy to slow tumor growth.^2,3,5^

The role of sterols as biomarkers extends beyond cancer studies, and sterol isomers can reveal key insights into the mechanisms of diseases. Smith–Lemli–Optiz syndrome (SLOS) and desmosterolosis are both disorders of cholesterol biosynthesis that cause prenatal malformations and often early postnatal lethality.^12−15^ The genetic cause of SLOS was revealed in 1993—29 years after it was first described—by a report finding highly elevated levels of 7-dehydrocholesterol (7-DHC) in patients with the disease.^12^ SLOS was found to result from a dysfunction in the generation of cholesterol from 7-DHC, its direct precursor via the Kandutsch–Russell biosynthetic pathway,^12,13^ a discovery that enabled patients with incorrect or unknown diseases to be accurately diagnosed with SLOS.^12^ Desmosterolosis is, likewise, caused by a mutation in the gene that encodes the enzyme responsible for converting desmosterol to cholesterol in the Bloch pathway, resulting in elevated levels of desmosterol in patients with the disease.^12,14,15^

Desmosterol and 7-DHC are carbon–carbon double-bond (C=C bond) isomers of each other, varying from each other only in the placement of their second C=C bonds at the 24 and 7 positions, respectively,^12^ but elevated presence of either isomer is indicative of very separate mutations, demonstrating the importance of the ability to differentiate C=C bond isomers. These discoveries have intensified the need for research into sterol metabolism and spurred the development of specific and accurate methods for studying nonpolar lipids, such as sterols.

Mass spectrometry (MS) has emerged as a powerful tool for lipidomic studies due to its high specificity and sensitivity in measuring spatial and temporal alterations in lipid composition. MS presents a promising approach for expanding the understanding of sterol metabolism; however, it operates on the principle of measuring mass-to-charge ratios (*m/z*) of ions, which can present difficulties when analyzing less ionizable molecules, such as nonpolar lipids. More easily ionizable lipids, such as glycerophospholipids, often dominate the mass spectrum, as they can be precharged or are more efficiently ionized in the electrospray. In contrast, nonpolar lipids (including squalene, cholesteryl esters, wax esters, triglycerides, and cholesterol), which account for over a third of the total lipids in human epidermal cells,^16^ cannot be ionized well, thus their species and isomeric forms are more challenging to detect by MS.

Gas chromatography–mass spectrometry (GC-MS) and high-performance liquid chromatography (HPLC) coupled with electrospray ionization mass spectrometry (ESI-MS) are the most prolific methods for the characterization of sterol lipids. In GC-MS, sterols are chemically modified to generate volatile derivatives that can then be ionized by either electron impact (EI) to produce informative fragments in the form of radical cations^17^ or chemical ionization (CI) to ionize the analyte with a gas such as ammonia;^18^ however, the temperature of the capillary GC column often exceeds 325 °C, which may cause lipid degradation, and EI is often less than ideal due to a lack of molecular ion information.^12^ Additionally, if the sterols are present in conjugated forms (e.g., sulfates and glucuronides), a hydrolysis step must be included to remove the conjugate moiety before sterol derivatization.^6,19,20^ HPLC-ESI-MS is more sensitive than GC-MS,^6^ but because tandem (MS/MS) analysis of sterol lipids by collision-induced dissociation (CID) is typically dominated by the fragment ions corresponding to the loss of water, HPLC-ESI-MS/MS is extremely reliant on optimizing chromatographic separation to match retention times to those of standards and provides limited information on the structural moiety.^19^

To enhance the ionization efficiency of nonpolar lipids, it is often necessary to use specific methods of ionization or modify the lipid structure by introducing a more ionizable group. Atmospheric-pressure chemical ionization (APCI),^21^ matrix-assisted laser dissociation ionization (MALDI),^22^ and direct analysis in real time (DART)^23^ have all been used to facilitate ionization for the analysis of nonpolar lipids with more effective ionization efficiencies than those seen with ESI.^24^ Likewise, derivatization methods targeting hydroxy^25,26^ and carboxyl^27^ groups were shown to facilitate the visualization of nonpolar lipids in MS. However, these methods do not provide C=C bond isomer resolution.

Recent advances in lipid methodologies have made it possible to analyze lipids at the isomer level using novel ion activation methods such as ozone-induced dissociation (OzID),^28^ ultraviolet photodissociation (UVPD),^29^ and radical-directed dissociation (RDD),^30^ as well as derivatization methods including Paternò–Büchi (PB),^31^ epoxidation,^32,33^ singlet oxygen (^1^ΔO_2_)–ene reactions,^34^*N*-Me aziridination,^35^ and light-catalyzed lipid double-bond cycloaddition.^36^ These methods represent important progress in lipid analysis, but not all of them are equally effective at improving the ionization efficiency of nonpolar lipids. Unionized compounds cannot be visualized by MS and thus it is valuable to have a method for isomer differentiation that is capable of simultaneously increasing ionization efficiency.

In this work, we report the *N*-H aziridination of the C=C bond in nonpolar lipids, which can be readily fragmented by CID to produce specific fragment ions for accurate C=C bond position determination. Moreover, *N*-H aziridination enhances the lipid ionization efficiency, further improving the sensitivity and accuracy of the nonpolar lipid analysis.

## Experimental Section

### Lipid Nomenclature

The shorthand lipid notations used in this work abide by the guidelines established by Liebisch and colleagues.^37^ The lipid class is denoted by its abbreviation, followed by the length of the *sn*-positional isomer fatty acyl chains separated by “/”, with the C=C bond position indicated in parentheses after the fatty acyl chain. Sterol lipid structures are also referred to by labeling the cyclopentanoperhydrophenanthrene skeleton rings A, B, C, and D, ring A being the cyclohexane containing carbon 1 and ring D being the cyclopentane.

### Materials and Reagents

Water (HPLC grade), methanol (MeOH, HPLC grade), acetonitrole (ACN, HPLC grade), isopropanol (IPA, HPLC grade), stigmasterol, ergosterol, and 7-dehydrocholesterol were purchased from Sigma-Aldrich (MO, USA). Hydroxylamine-*O*-sulfonic acid (HOSA) was purchased from Combi-Blocks (CA, USA). Hexafluoro-2-propanol (HFIP), cholesterol, and β-sitosterol were purchased from Fisher Scientific (NH, USA). Pyridine was purchased from Millipore Sigma (MA, USA). Bis[rhodium(α,α,α′,α′-tetramethyl-1,3-benzenedipropionic acid)] (Rh_2_(esp)_2_) was from AmBeed (IL, USA). All solvents and chemicals were used without further purification.

### Aziridination of Nonpolar Lipid Standards

Lipid aziridination catalyzed by Rh_2_(esp)_2_ was performed as previously reported.^38^ Briefly, lipid standards were dissolved in HFIP to achieve a final concentration of 10 mM. HOSA (1.5 equiv of the C=C bond), pyridine (3 equiv), and Rh_2_(esp)_2_ (5 mol %) were added, and the reaction mixture was stirred at room temperature for 3 h. Reaction mixtures were diluted 20-fold with MeOH/H_2_O (2:1 v/v) prior to analysis.

### Mass Spectrometry

Lipid analysis was performed on a Q-Exactive Plus hybrid quadrupole orbitrap mass spectrometer from Thermo Fisher Scientific (San Jose, CA). NanoESI emitters were made from borosilicate glass tubing with filament on a P-100 micropipet puller, both from Sutter Instrument Company (Novato, CA), using the following parameters: heat 545, pull 20, velocity 25, time 250, and pressure 500. Samples were analyzed in the positive ion mode. The following MS parameters were set for data acquisition: spray voltage 1.3 kV, capillary temperature 275 °C, S-lens RF level 60.0, and *m*/*z* range 100–500. Higher-energy collisional dissociation (HCD) was used to obtain tandem mass spectra at a normalized collision energy (NCE) of 10–55.

### Lipid Extraction from Tissue

Lipids were extracted using a modified protocol reported previously.^39^ Briefly, 20 mg of tissue was homogenized in 90 μL of deionized water. After the addition of 810 μL of chloroform/methanol (1:2 v/v), the sample was vortexed for 1 min and incubated for 1 h at 4 °C. After adding 300 μL of chloroform and 400 μL of deionized water and vortexing for 1 min between each addition, the samples were centrifuged for 5 min at 12 000 rpm. Samples were dried under a nitrogen stream and stored at −80 °C until analysis. For aziridination, moles of lipid extracted were estimated using 600 g/mol. The extracted lipids were resuspended in 100 μL of dichloromethane, then mixed with HOSA (7.5 equiv), pyridine (15 equiv), Rh_2_(esp)_2_ (25 mol %), and HFIP to a final volume of 500 μL. The reaction mixture was stirred at room temperature overnight and diluted 5× with methanol/water (2:1 v/v) for analysis.

### Biological Sample HPLC Analysis

A Vanquish HPLC system (Thermo Fisher Scientific) coupled with an Orbitrap Velos Pro mass spectrometer (Thermo Fisher Scientific) was deployed for the LC-MS analysis of healthy and cancerous mouse prostate samples. A solvent system of ACN/water (60:40 v/v) (solvent A) and IPA/ACN (90:10 v/v) (solvent B), both containing 0.1% formic acid and 10 mM ammonium formate, was used. An aliquot of 5 μL of the sample was injected into the Accucore C30 column (Thermo Fisher Scientific, C30, 2.1 mm × 150 mm, 2.6 μm). A flow rate of 0.200 mL/min and a column temperature of 40 °C were used. The elution gradient was modified from a previously reported method^40^ and was as follows: 30%–43% B at 0–5 min, 43–50% B at 5–5.1 min, 50–70% B at 5.1–14 min, 70–99% B at 14.1–21 min, 99% B at 21–24 min, 99–30% B at 24–24.1 min, and 30% B at 24.1–33 min. Data were acquired with a top 3 data dependent acquisition (DDA) method using a *m*/*z* range of 100–1000, a full MS resolution of 30 000, a MS/MS resolution of 15 000, and HCD with an NCE of 35. Additionally, CID with an NCE of 40 was used to visualize triacylglycerol (TG) diagnostic ions that were not produced under HCD.

### Data Analysis

MS data were processed using the Xcalibur Qual Browser (Thermo Fisher Scientific). Biological sample data were processed by extracting ion intensities of lipids of interest using a 30 s window centered on the elution peak and normalizing over the total ion current within that window and grams of tissue sample used. A heatmap of the normalized biological data was generated using MetaboAnalyst.^41^

## Results and Discussion

### Aziridination-Assisted Mass Spectrometry of Nonpolar Lipids

The aziridination of olefin results in the formation of an aziridine, which is a highly valuable three-membered nitrogen-containing ring due to its remarkable versatility and importance in various applications, including the synthesis of complex natural products, pharmaceuticals, and materials science. Our previous work demonstrated the use of lipid *N*-H aziridine to introduce isobaric mass tags for lipid-accurate quantification.^30^ In this work, we focus on the analysis of nonpolar lipids, such as sterols. We activate C=C bonds in nonpolar lipids by aziridination, which allows for the opening of the aziridine ring upon CID and the generation of characteristic fragments that enable the determination of the C=C bond position. Moreover, the introduction of nitrogen into nonpolar lipids substantially enhances the ionization efficiency, enabling the clear detection of intact lipids and their characteristic fragments for the C=C bond location by MS/MS.

**Scheme 1:**
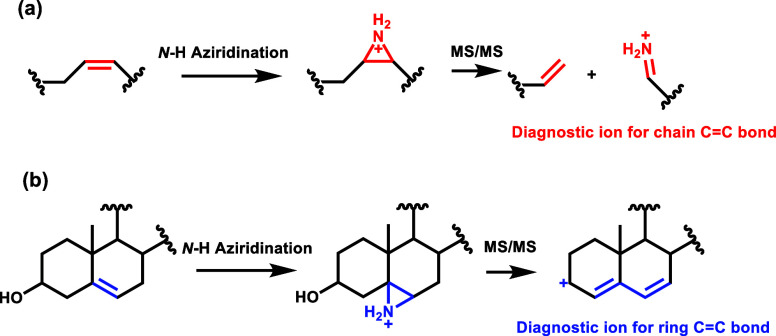
Mechanism of *N-*H Aziridination Coupled with CID-MS/MS for (a) Chain C=C Bond and (b) Ring C=C Bond Identification

Nonpolar lipids, such as sterols, often contain two types of C=C bonds. Our investigation revealed that activation of these C=C bonds by aziridination resulted in distinct fragmentation patterns for the chain C=C bonds and ring C=C bonds upon CID. Scheme 1 illustrates the mechanism of aziridination in conjunction with MS/MS analysis for the two types of C=C bonds present in nonpolar lipids. Upon CID-MS/MS, the aziridinated chain C=C bond undergoes fragmentation, yielding ring-opening products, where the imine serves as the diagnostic ion (Scheme 1a). In contrast, the aziridinated ring C=C bond produces a diene with an allylic carbocation (Scheme 1b). The conjugated system provides stabilization for the allyl carbocation, resulting in a distinct fragmentation for this type of bond.

**Figure 1:**
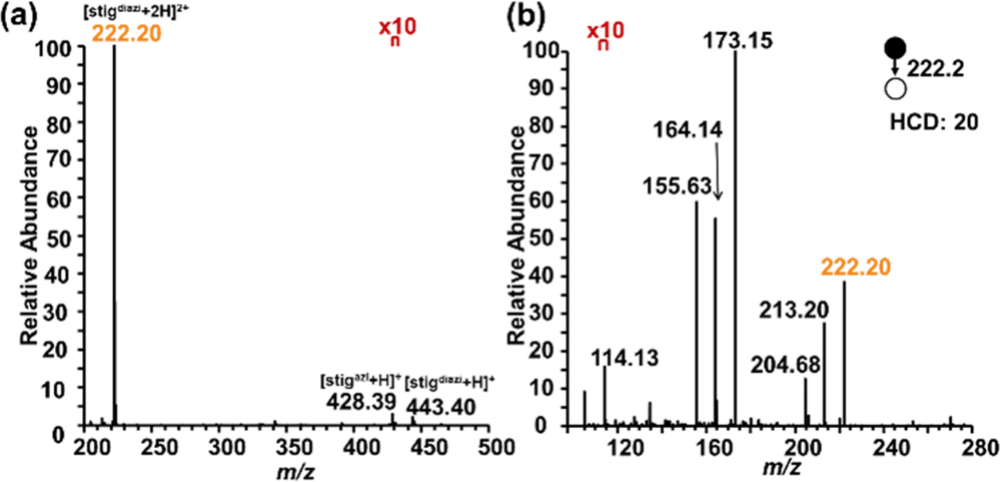
(a) Mass spectrum of stigmasterol after aziridination, where its diprotonated diaziridinated, monoprotonated monoaziridinated, and monoprotonated diaziridinated products are detected at *m*/*z* 222.20, 428.39, and 443.40, respectively. (b) CID tandem mass spectrum of the doubly charged stigmasterol diaziridine product.

We chose stigmasterol as a model lipid to study these two fragmentations because it contains two types of C=C bonds, with one located in the side chain at position 17 and the other located in the ring backbone. Stigmasterol can be aziridinated using HOSA, a nitrogen source, and catalyzed by the Du Bois catalyst Rh_2_(esp)_2_ in HFIP. After the formation of stigmasterol aziridine, a 15.0109 Da mass shift (corresponding to the addition of an *N*-H group) occurs at each C=C bond. This results in the formation of a doubly charged aziridinated stigmasterol species, which can be detected at *m*/*z* 222.2036, though its singly and doubly derivatized products were also detected in much lower abundances (Figure 1a). In contrast, the unreacted stigmasterol molecule is not easily detected by MS (data not shown).

**Scheme 2:**
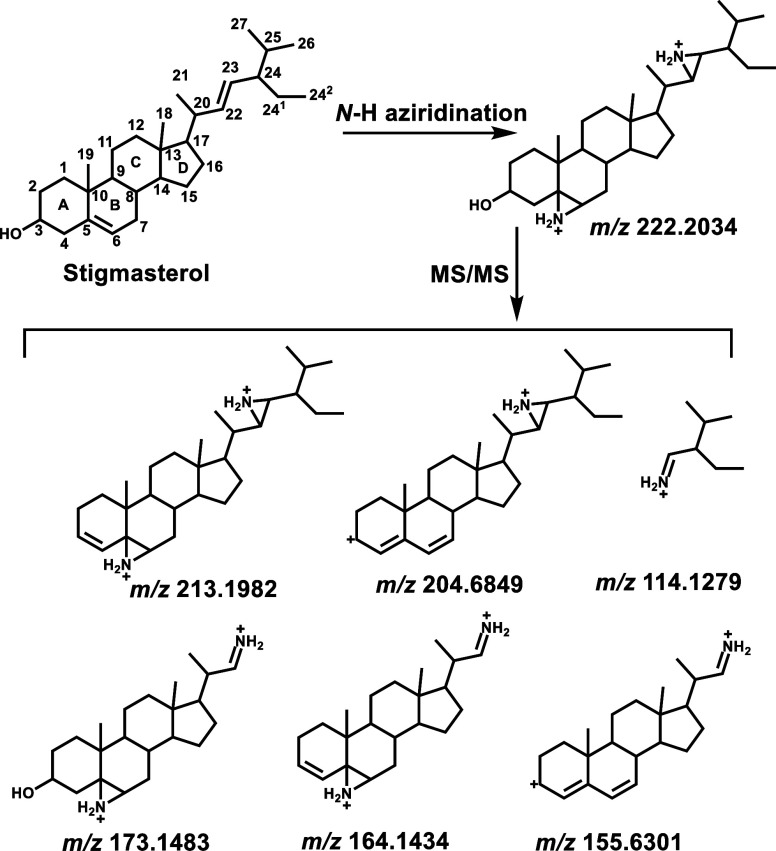
*N-*H Aziridination of Stigmasterol Followed by CID-MS/MS to Produce Specific Fragment Ions for Locating C=C Bonds in the Side Chain and Ring Backbone

**Scheme 3:**
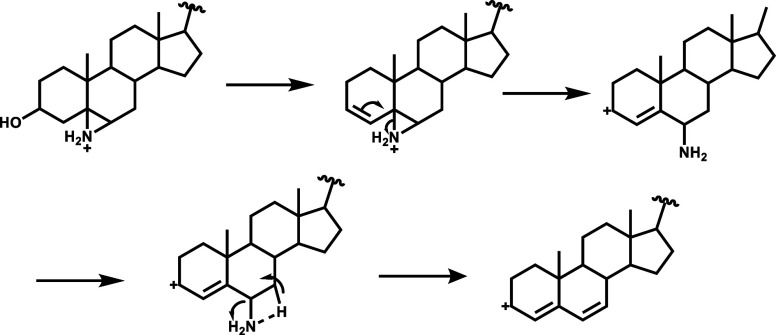
Proposed Pathway for Fragmenting Ring Aziridine to Form a Conjugated Allylic Carbocation upon CID

Fragmenting aziridinated stigmasterol generated the ions at *m*/*z* 213.1984, 204.6852, 173.1489, 171.1517, 164.1436, 155.6303, and 114.1279 due to water elimination and fragmentation of side chain aziridine and backbone aziridine (Figure 1b, Scheme 2). The side chain aziridine ring can be cleaved from both sides by CID, resulting in characteristic fragments at *m*/*z* 114.1279 and 173.1483. These fragments are indicative of the presence of the C=C bond at position 22 in stigmasterol. Dehydration resulted in the loss of the hydroxyl group on the A ring, as evidenced by the *m*/*z* values of 213.1984 and 164.1436. This loss facilitated the generation of a conjugated allylic carbocation on the A ring, observed at *m*/*z* 204.6852 and 155.6303. Scheme 3 illustrates the proposed pathway for this process.

**Figure 2:**
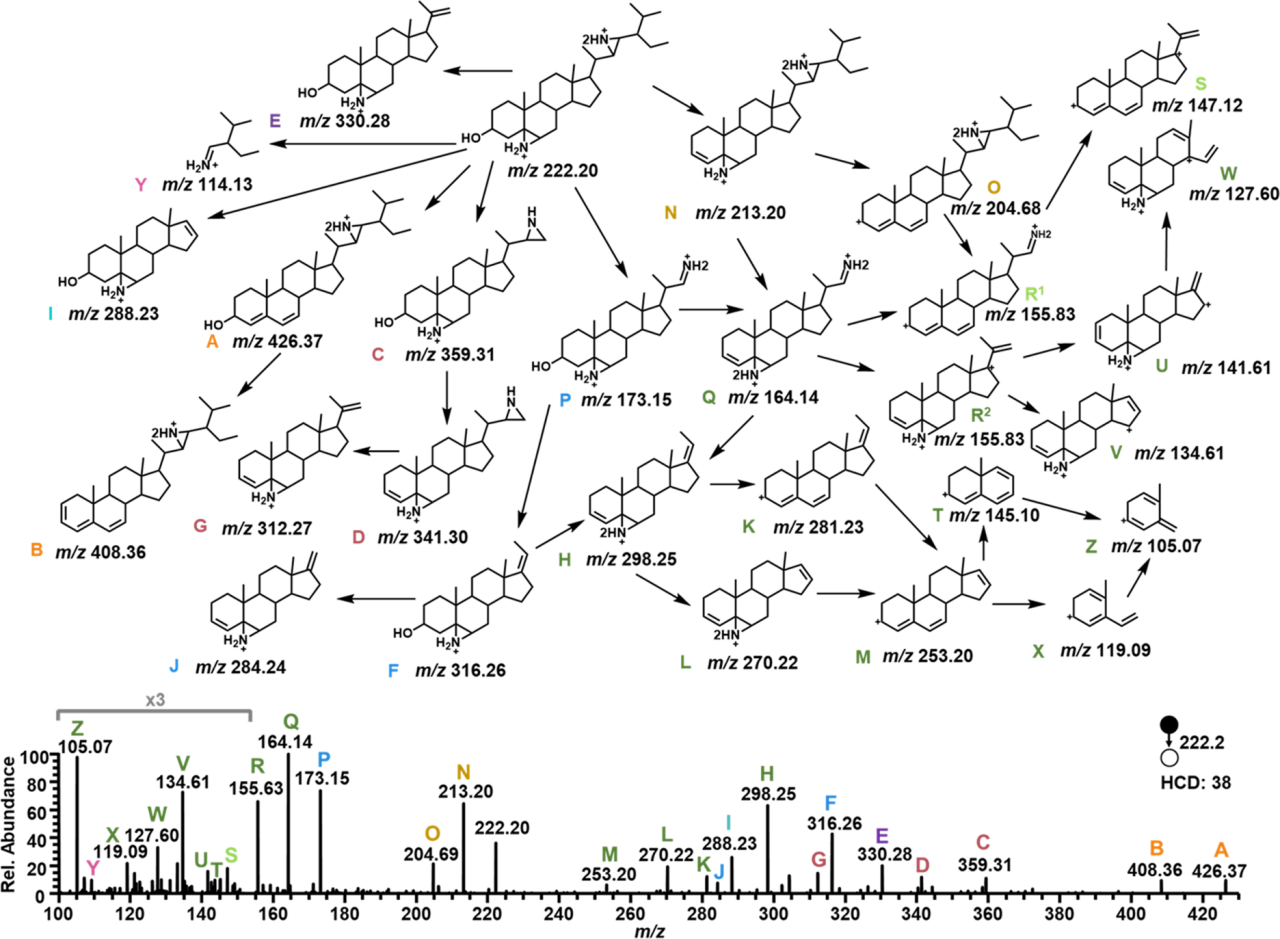
Fragmentation pathway of the diprotonated diaziridinated stigmasterol product and corresponding labeled MS/MS spectrum at *m*/*z* 222.2.

Aziridination can also assist the identification of the sterol backbone. A fragmentation pathway of the protonated stigmasterol aziridine was shown in Figure 2. High resolution data from HCD and MS^3^, MS^4^, and MS^5^ were used to confirm the fragment structure determination and the order of functional group fragmentation. There was no obvious regioselectivity between the water loss in the backbone and the side chain aziridine ring opening, as evidenced by the two fragmentation pathways observed in the MS^2^ spectrum: one pathway from the diaziridinated stigmasterol product ions at *m*/*z* 222.20 to ions at *m*/*z* 213.20 (N) showing water loss was before side chain aziridine ring opening, and the other pathways from the diaziridinated stigmasterol product ions at *m*/*z* 222.20 to ions at *m*/*z* 173.15 (P) showed the opposite process (Figure 2). Altogether, 26 fragments were identified. Beyond the diagnostic ions (P, Y, and R^1^), novel fragments due to aziridine were observed at *m*/*z* 426.3719, 408.3615, 359.3041, 341.2946, 330.2781, 316.2626, 312.2673, 298.2524, 288.2315, 270.2207, 213.1980, 204.6846, 173.1484, 164.1432, 141.6144, 134.6066, 127.5989, and 114.1280. Fragmentation of the stigmasterol diaziridine is primarily generated through pathways stemming from three ions: the loss of water at *m*/*z* 213.20 (N), the cleavage of the aziridine on the 17-position chain at *m*/*z* 173.15 (P), and the fragmentation of the 17-position chain that leaves the aziridine intact at *m*/*z* 359.31 (C). The pathway stemming from N, labeled in gold, results from the loss of the aziridine on the B-ring to generate an ion at *m*/*z* 204.68 (O). Further fragmentation cleaves the remaining aziridine (R^1^), which is then broken off as the 17-position chain fractures (S). The generally most abundant ions are spawned from fragmentation of diagnostic ion *m*/*z* 173.15 (P), labeled in blue, which can then fragment by one of two mechanisms: degradation of the chain (F) or loss of water (Q). In the breakdown of the 17-position chain, the B-ring aziridine was observed to remain intact (H and J). Fragmentation from Q, likewise, could converge with the singly charged pathway (dark green) from F as the imine of Q is fragmented off, generating H at *m*/*z* 298.25. H can then lose both the B-ring aziridine and the remnants of the 17-position chain in any order (K and L) before the sterol ring backbone is broken down (M, T, X, and Z). Two additional paths stem from Q, both by the generation of a fragment at *m*/*z* 155.83: the convergence with the N pathway (R^1^, light green) or the generation of R^2^ by losing either the B-ring or chain aziridines, respectively. The pathway from R^2^, labeled in dark green, generates a fragment with a unique feature; once the 17-position chain breaks down to *m*/*z* 127.60, the ring can then break down while the B-ring aziridine stays intact (W), which is a fragmentation behavior that was otherwise not observed. The ions produced from the C pathway (red) are generally less abundant, as its mechanism includes the terminal end of the 17-position chain fragmenting off, while the chain aziridine remains intact but loses its charge. The hydroxy group is then fragmented off in the loss of water (D), followed by the loss of the 17-chain aziridine, generating a fragment at *m*/*z* 312.27 (G).

### Identification of Sterol C=C Bond Positional Isomers

**Figure 3:**
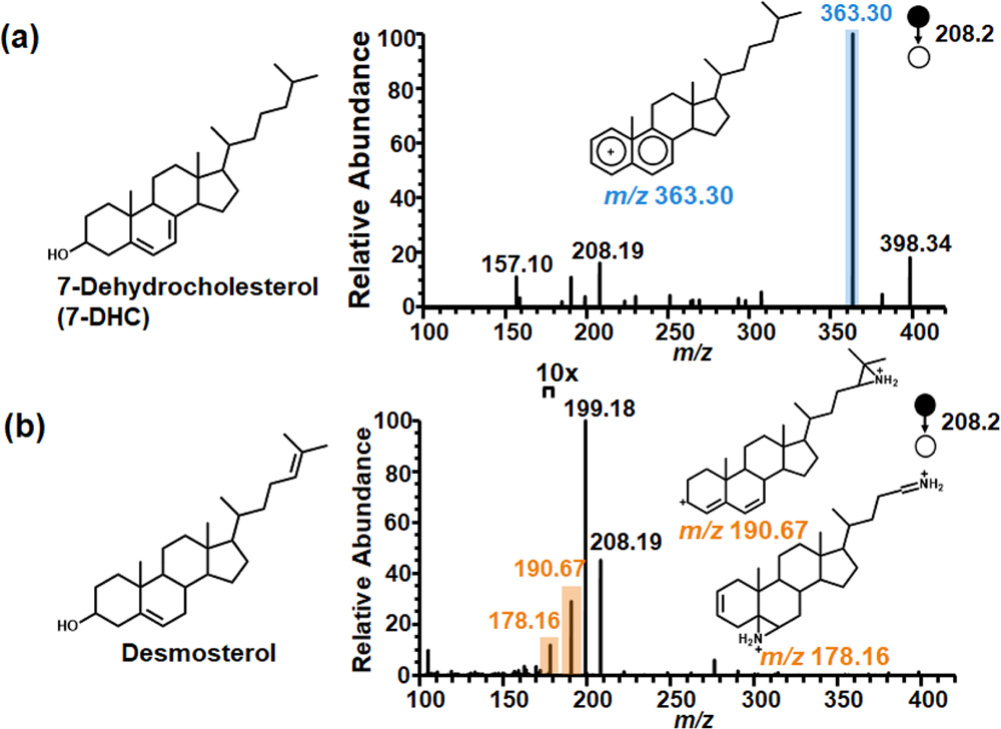
MS/MS fragmentation of doubly charged diaziridinated (a) 7-DHC and (b) desmosterol distinguishes the isomers by producing the diagnostic ions at *m*/*z* 363.30 for 7-DHC and diagnostic peaks at *m*/*z* 190.67 and 178.16 for desmosterol.

7-DHC and desmosterol are a pair of C=C bond positional isomers. They have the same molecular mass of 384.3392 Da, but the C=C bonds are located on either the 17-position chain or the backbone ring. After aziridination of the two isomers, their doubly charged diaziridine products were shown at *m*/*z* 208.1875. CID-MS/MS analysis of the isomers yielded two distinct spectra, as shown in Figure 3. Fragmentation of 7-DHC aziridine preferentially generated a singly charged fragment ion at *m*/*z* 363.3043, while desmosterol produced characteristic fragment ions at *m*/*z* 190.6690 and 178.1588. The observed fragmentation patterns provided clear differentiation between the chain C=C bond and the backbone C=C bond (Figure 3). We also applied aziridination-assisted MS analysis to β-sitosterol and ergosterol (Figure S1 and S2). Fragmentation of their lipid aziridines produced expected diagnostic ions. These findings underscore the ability of aziridination-assisted MS analysis to characterize nonpolar lipids and discern positional isomers of sterol C=C bonds.

### Limit of Detection and Relative Quantification of a Nonpolar Lipid

**Figure 4:**
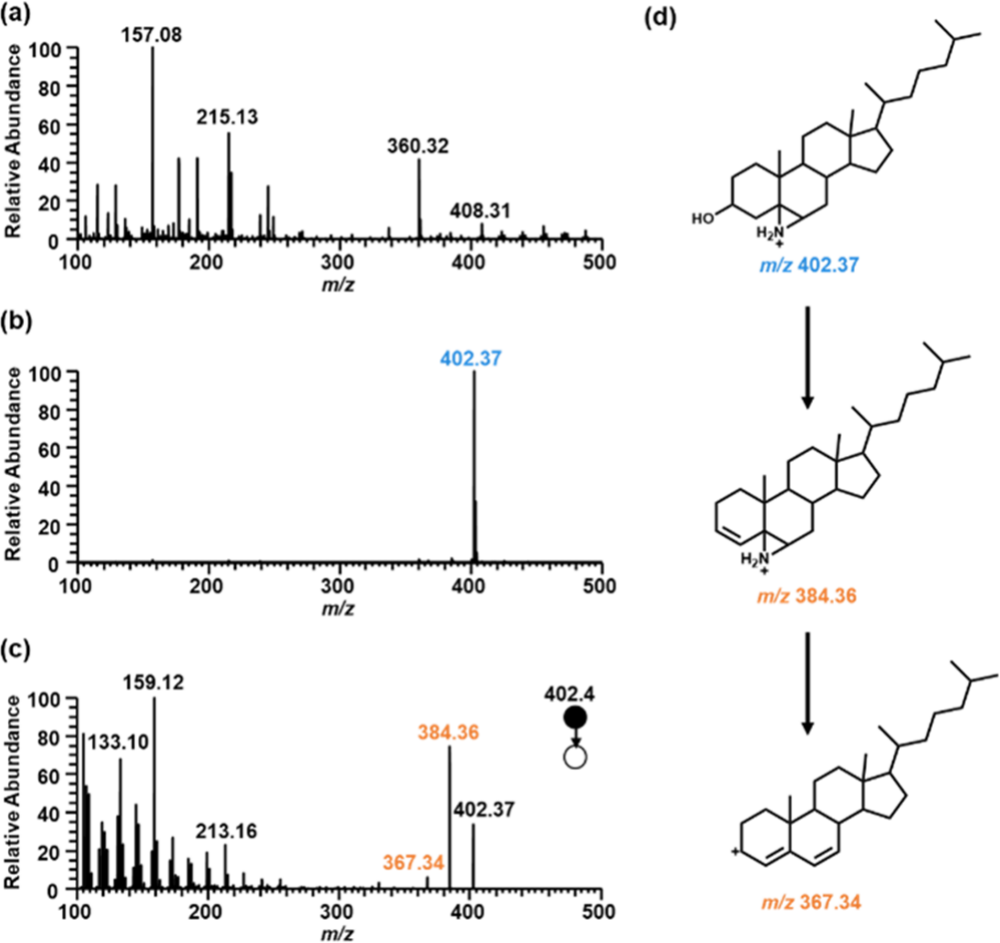
Mass spectra of (a) unmodified cholesterol, (b) aziridinated cholesterol, and (c) aziridinated cholesterol MS/MS at *m*/*z* 402.37. (d) Fragmentation pathway to form ions at *m*/*z* 384.36 and 367.34.

Aziridination of nonpolar lipids dramatically improves the sensitivity and allows the identification of nonpolar lipids at low abundance. For example, ESI-MS analysis failed to detect unmodified cholesterol prepared in methanol and water (2:1 v/v) containing 0.1% formic acid, with all of the observed peaks in the spectrum being attributed to background/solvent signals (Figure 4a). In contrast, analysis of aziridinated cholesterol yielded the protonated aziridine peak at *m*/*z* 402.37 in high abundance (Figure 4b). MS/MS analysis of the aziridine produced diagnostic ions at *m*/*z* 384.36 and 367.34 (Figure 4c and d). A “fingerprint” series of ions of the sterol backbone fragments were also observed (Figure 4c), and the complete fragmentation pathway is shown in the Supporting Information (Figure S3). The detection limit of cholesterol was assessed using aziridination-assisted ESI-MS at various concentrations, and it was found that a concentration of 10 nM was detectable through direct infusion analysis (Figure S4).

**Figure 5:**
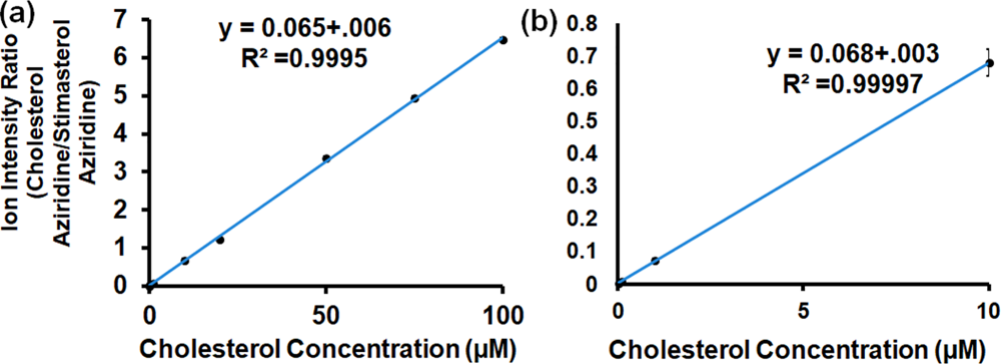
Relative quantification of cholesterol using aziridination-assisted MS showing (a) cholesterol concentrations from 10 nM to 100 μM and (b) lower concentrations from 10 nM to 10 μM.

Relative quantification of cholesterol was conducted using stigmasterol aziridine as a reference (Figure 5). The concentration of reference stigmasterol aziridine was maintained at 10 μM, while concentrations of cholesterol were prepared at 100 μM, 50 μM, 20 μM, 10 μM, 1 μM, 100 nM, 50 nM, and 10 nM. The calibration curve was constructed by plotting the ion intensity ratio of cholesterol aziridine ions to stigmasterol aziridine ions against the original concentration of cholesterol prior to aziridination. The ion intensity ratio was calculated by extracting the area under the curve of cholesterol aziridine (*m*/*z* 402.37) and doubly charged diaziridinated stigmasterol (*m*/*z* 222.20). A linear relationship was observed, with a good *R*^2^ value of 0.99953 for the full concentration range (Figure 5a) and that of 0.99997 for the lower concentration range (10 nM to 10 μM) (Figure 5b).

### Application of Aziridination-Assisted MS Analysis in the Characterization of Nonpolar Lipids from Cancerous Mouse Prostate Samples

Prostate cancer has been associated with the upregulation of cholesterol and its derivatives, which sustain increased cell proliferation,^2^ and alterations in the lipidome have been suggested as a potential biomarker for the diagnosis of prostate cancer.^5^ To demonstrate the applicability of lipid aziridination for the MS-based characterization of nonpolar lipids in biological samples, nonpolar lipids were extracted from healthy and cancerous mouse prostates, aziridinated, and analyzed by HPLC-MS. Identities of detected lipids were confirmed by MS/MS.

**Figure 6:**
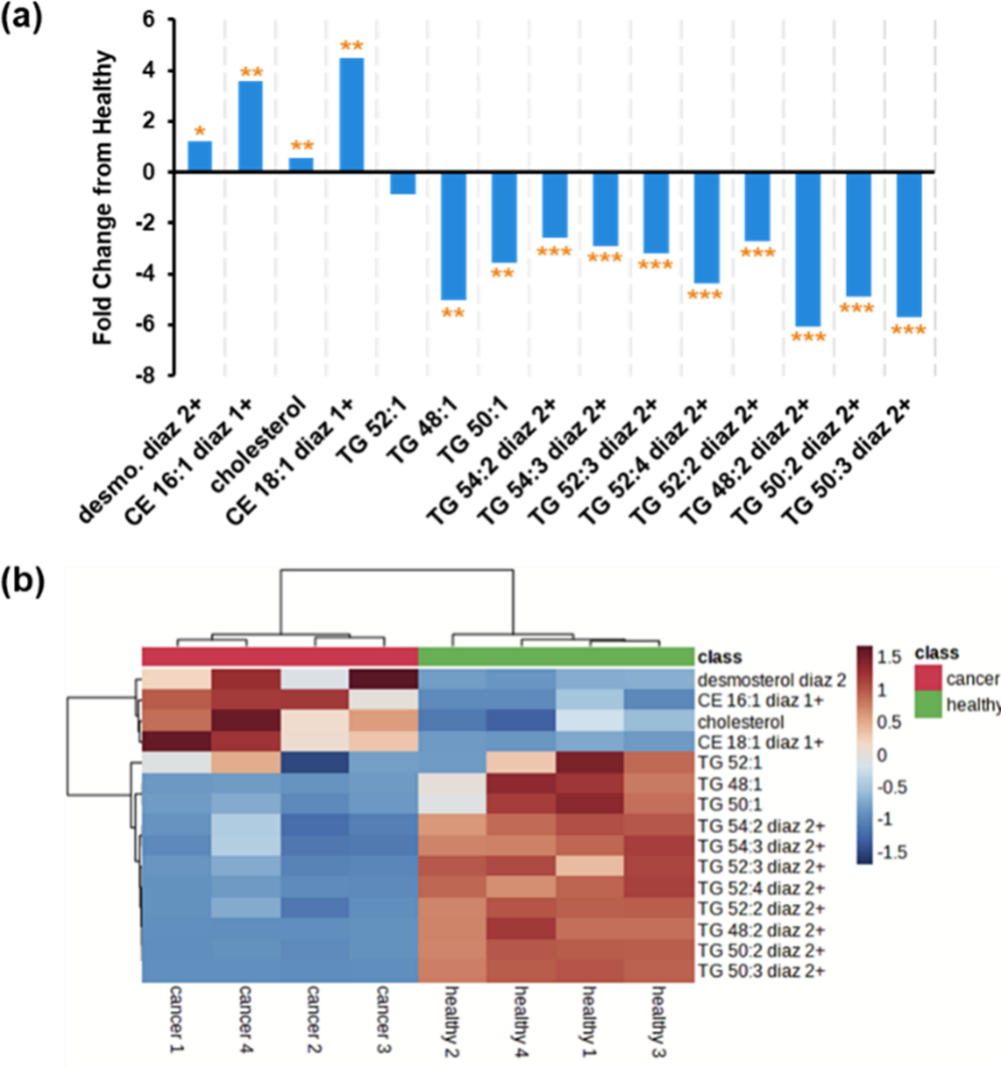
(a) Fold changes in the ion abundances per gram of tissue of nonpolar lipid aziridines from mouse prostate samples. The two-tailed Student’s *t* test was used to evaluate the differences between the healthy and tumorous samples for statistical significance (*p< 0.05, **p< 0.01, ***p< 0.001). (b) Heatmap of the normalized ion intensities of nonpolar lipid aziridines in healthy and tumorous mouse prostate samples.

Nonpolar lipids including cholesterol (Figure S5), desmosterol, cholesterol ester, and TG lipids were successfully visualized with isomer resolution in full MS (Table S1). Under optimized conditions, lipids containing multiple C=C bonds most abundantly produced doubly charged diaziridine ions. We found that sterols and TGs presented distinct trends in the two groups. An upregulation of cholesterol in the cancerous samples was detected; the average abundance of cholesterol in the cancerous samples increased 0.57-fold, while the abundances of CE 16:1 and CE 18:1 had fold increases of 3.56 and 4.49, respectively (Figure 6a and b). Although sterols overall experienced an increase in abundance in the cancerous samples, the TGs had fold decreases of as much as 6.06 and were less abundant than those in healthy samples for all ions analyzed. The fold changes for all analyzed lipids except TG 52:1 were found to be statistically significant by the Student’s *t* test at a 95% confidence level. Hierarchical cluster analysis of healthy and cancerous samples mouse prostate samples using nonpolar lipids indicates these lipids can differentiate the two groups unambiguously (Figure 6b).

## Conclusions

We have developed a method for simultaneously enhancing ionization efficiency and determining the C=C bond position in nonpolar lipids. The method uses Rh_2_(esp)_2_ to catalyze the aziridination of C=C bonds with HOSA in HFIP and subsequent collision-induced dissociation to generate characteristic fragment ions for the identification of C=C bond positions and fingerprint ions for the characterization of backbone. The method has a significant impact on the ionization efficiency, with a limit of detection of 10 nM. We have shown that the fragmentation pathways of chain C=C bonds and ring C=C bonds are distinct, and we have successfully differentiated between isomers 7-DHC and desmosterol, synthetic precursors of cholesterol. Furthermore, we have demonstrated the feasibility of the approach in complex biological samples by coupling the aziridination of nonpolar lipids with HPLC-MS using mouse prostate cancerous tissues. This method has enabled clear visualization of nonpolar lipids with upregulation of sterols and downregulation of TGs in cancerous prostate tissues. The high efficiency and specificity of aziridination-assisted MS analysis make it highly suitable for broad applications in nonpolar lipid studies.

## Supporting Information Available

MS analysis of aziridinated β-sitosterol, the MS analysis of aziridinated ergosterol, fragmentation pathway of aziridinated cholesterol, limit of detection of the aziridination-assisted MS analysis of cholesterol, and nonpolar lipid aziridines identified from mouse prostate samples (PDF)
