# Supplementary material for: Aziridination-Assisted Mass Spectrometry of Nonpolar Sterol Lipids with Isomeric Resolution
Source: J Am Soc Mass Spectrom. 2023 Jul 31;34(9):1998–2005. doi: 10.1021/jasms.3c00161 (PMC10863044; doi:10.1021/jasms.3c00161)
Supplement: Supplementary file 1 — js3c00161_si_001.pdf [file js3c00161_si_001.pdf]

Supporting Information

Aziridination-Assisted Mass Spectrometry of Nonpolar Sterol Lipids with Isomeric Resolution

Erin Hirtzel,[1] Madison Edwards,[1] Dallas Freitas,[1] Ziying Liu,[2] Fen Wang,[2] Xin Yan[1,]*

[1]Department of Chemistry, Texas A&M University, College Station, TX, 77843
[2]Center for Translational Cancer Research, Texas A&M University, Houston, TX, 77030

Corresponding Author
*Xin Yan: xyan@tamu.edu

Table of Content

S1. Aziridination-assisted MS analysis of β-sitosterol

S2. Aziridination-assisted MS analysis of ergosterol

S3. Fragmentation pathway of cholesterol after aziridination

S4. Limit of detection of cholesterol using aziridination-assisted MS analysis

S5. Analysis of nonpolar lipids extracted from mouse prostates

S1. Aziridination-assisted MS analysis of β-sitosterol

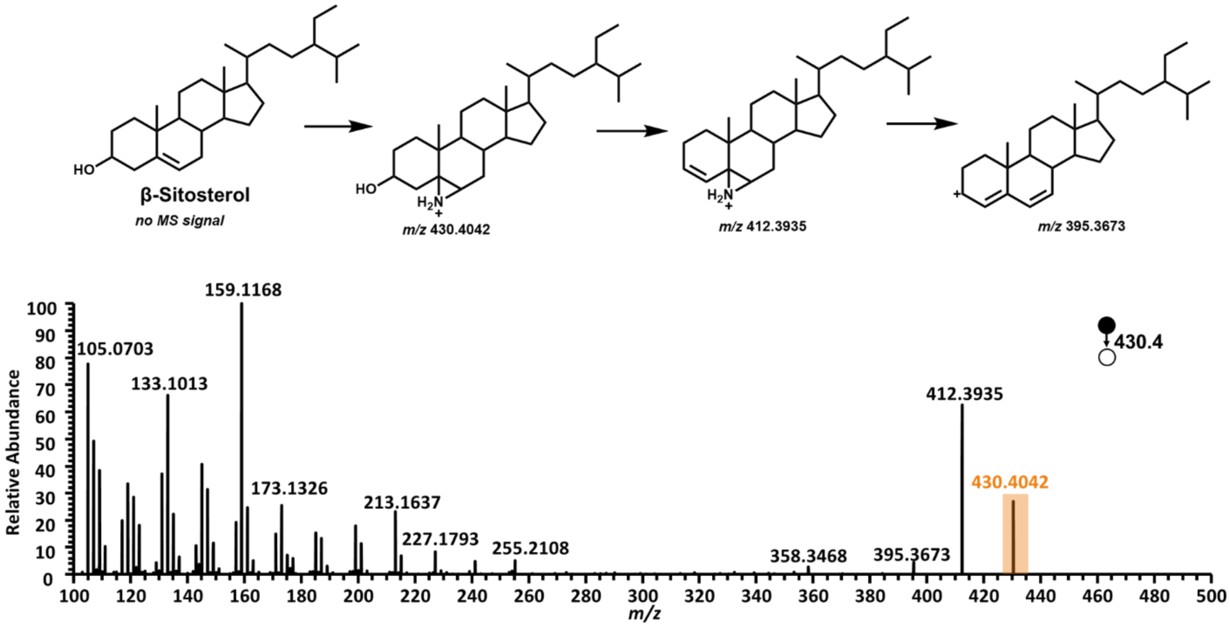

Figure S1. Tandem mass spectrum of β-sitosterol aziridine (*m/z* 430.4043) through aziridination-assisted MS analysis of β-sitosterol.

S2. Aziridination-assisted MS analysis of ergosterol

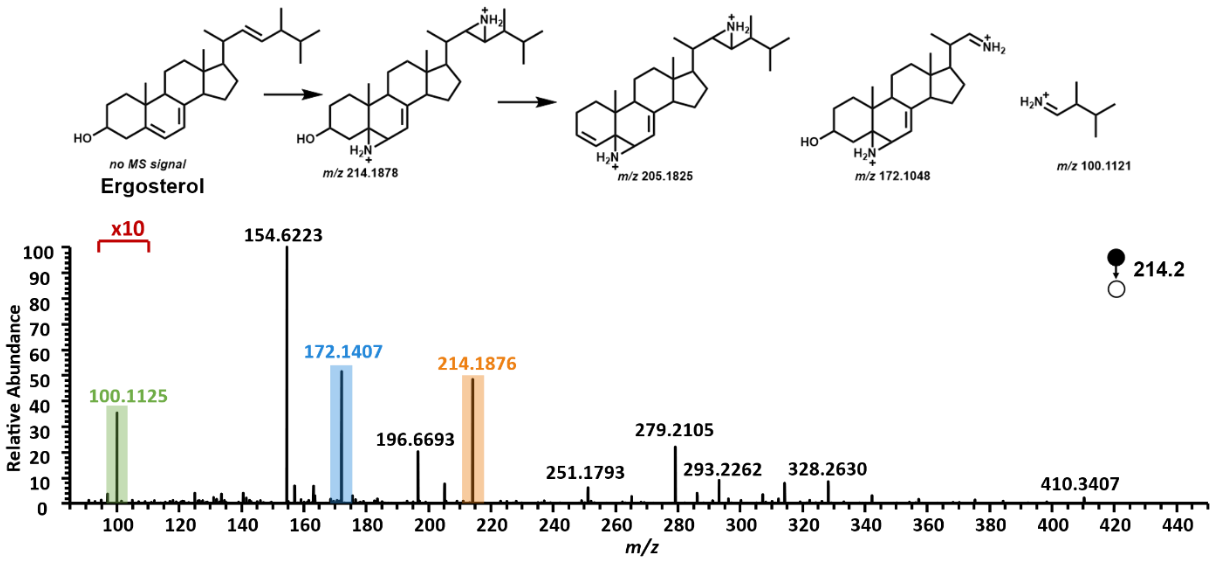

Figure S2. Tandem mass spectrum of ergosterol aziridine (*m/z* 214.1877) through aziridination-assisted MS analysis of ergosterol.

S3. Fragmentation pathway of cholesterol after aziridination

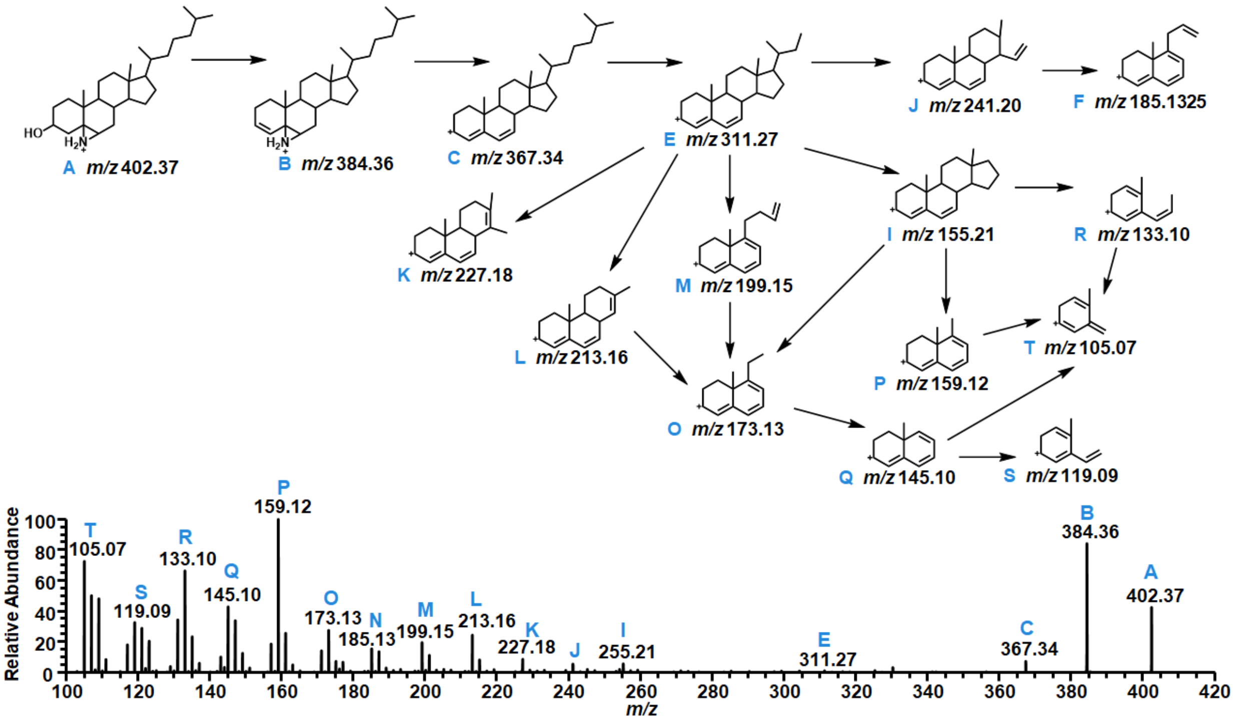

Figure S3. Fragmentation pathway of cholesterol aziridine at *m/z* 402.37 with annotated MS/MS spectrum.

S4. Limit of detection of cholesterol using aziridination-assisted MS analysis

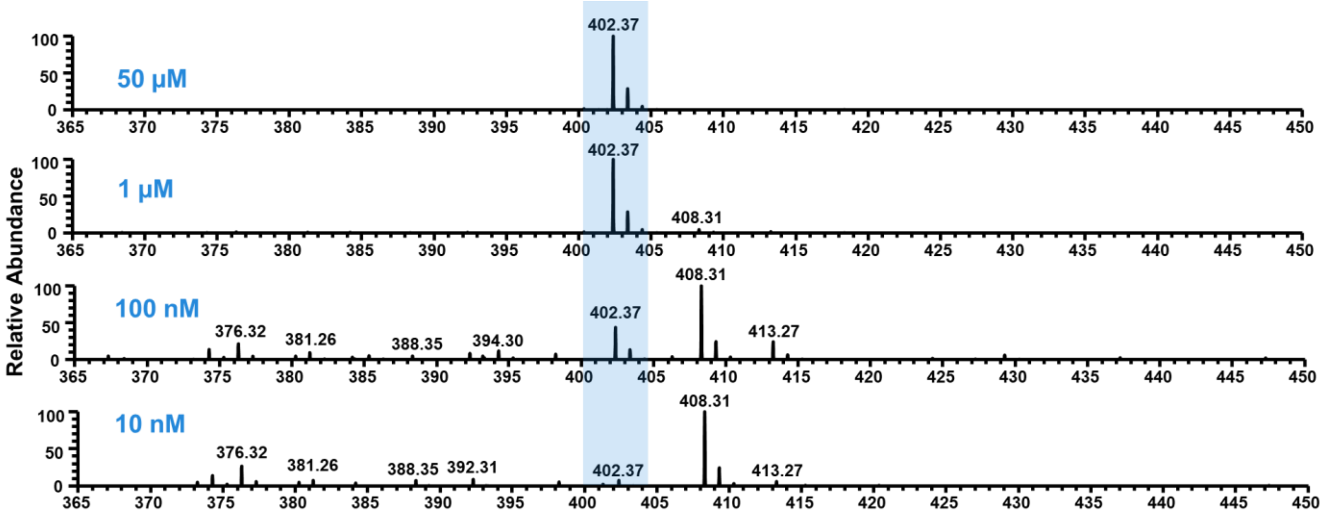

Figure S4. Limit of detection of cholesterol after aziridination (*m/z* 402.37). Cholesterol was analyzed from 50 µM to a limit of 10 nM.

S5. Analysis of nonpolar lipids extracted from mouse prostates

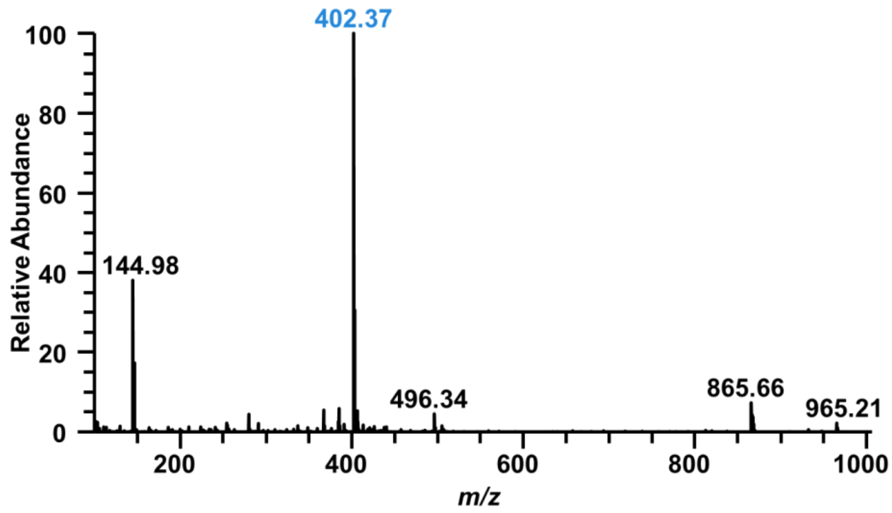

Figure S5. Full mass spectrum of lipid extract from mouse prostates obtained at the elution time of the cholesterol aziridine product (*m/z* 402.37).

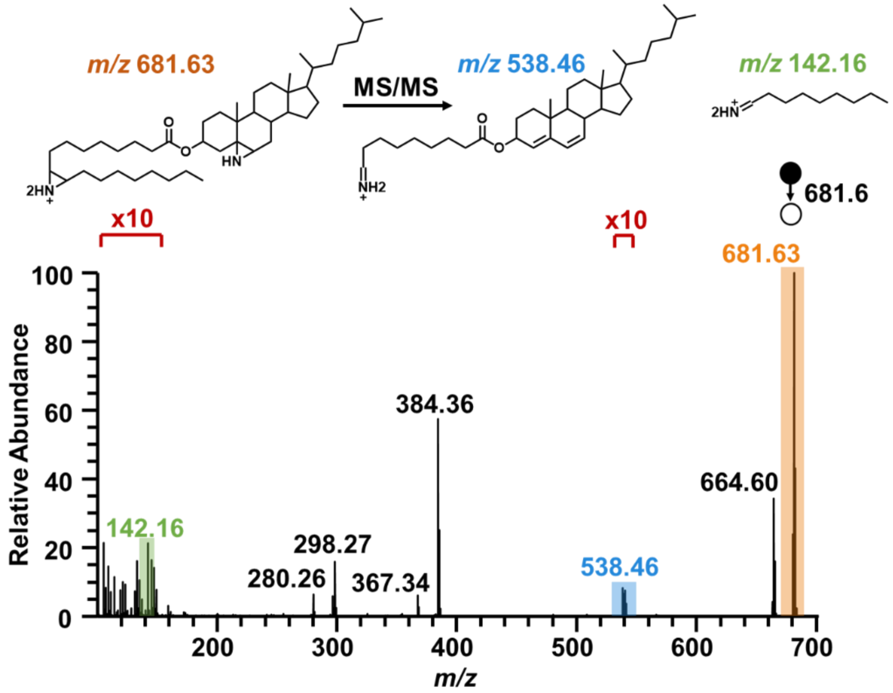

Figure S6. Tandem mass spectrum of CE 18:1 in cancerous mouse prostate sample. C=C double bonds position diagnostic ions are highlighted in green and blue at *m/z* 142.16 and *m/z* 538.46, respectively.

Table S1. List of nonpolar lipid aziridines, their most abundantly detected m/z, and their retention times.

lipid	charge	*m/z*	C=C diagnostic ions	C=C position	RT (min)
cholesterol	1+	402.37	-	-	5.38
desmosterol diaz	2+	208.19	178.2	-	1.77
CE 16:1 diaz	1+	653.6	114.1, 538.5	Δ9	9.37
CE 18:1 diaz	1+	681.62	142.2, 538.5<br>114.1, 566.5	Δ9<br>Δ11	10.94
TG 48:1	1+	820.74	694.6<br>708.6<br>722.6	n-9<br>n-8<br>n-7	19.59
TG 48:2 diaz	2+	417.37	354.3<br>361.3<br>368.3<br>375.3<br>382.3	n-9<br>n-8<br>n-7<br>n-6<br>n-5	9.96
TG 50:1	1+	848.77	708.6<br>722.7<br>750.7<br>764.7	n-10<br>n-9<br>n-7<br>n-6	20.17
TG 50:2 diaz	2+	431.39	361.3<br>368.3<br>375.3<br>382.3<br>389.3	n-10<br>n-9<br>n-8<br>n-7<br>n-6	11.04
TG 50:3 diaz	2+	430.38	374.3<br>381.3<br>388.3	n-8<br>n-7<br>n-6	10.71
TG 52:1	1+	876.8	736.6<br>750.7<br>778.7<br>792.7	n-10<br>n-9<br>n-7<br>n-6	21.07
TG 52:2 diaz	2+	445.4	375.3<br>382.3<br>389.3<br>396.3	n-10<br>n-9<br>n-8<br>n-7	12.13
TG 52:3 diaz	2+	444.39	381.3<br>388.3<br>395.3<br>402.3	n-9<br>n-8<br>n-7<br>n-6	11.84
TG 52:4 diaz	2+	443.39	380.3, 381.3<br>394.3<br>401.3<br>408.3<br>718.6<br>732.6<br>760.6	n-9<br>n-7<br>n-6<br>n-5<br>n-11-azi<br>n-10-azi<br>n-8-azi	11.55
TG 54:2 diaz	2+	459.42	389.3<br>396.3<br>403.4<br>410.4	n-10<br>n-9<br>n-8<br>n-7	13.79
TG 54:3 diaz	2+	458.41	388.3<br>395.3<br>402.3<br>409.4<br>416.4	n-10<br>n-9<br>n-8<br>n-7<br>n-6	13.51

Terminology:

The classification of lipids as “polar” and “nonpolar” is using generally accepted definitions in lipidomics. In the field of lipidomics, lipids are classified into the categories of polar and nonpolar by their overall hydrophobicity. Lipids that are generally soluble in extremely nonpolar solvents such as hexane (including TGs and steroids) are classified as nonpolar.[1]

REFERENCE

(1) X. Han, *Lipidomics: Comprehensive mass spectrometry of lipids*, John Wiley & Sons, Hoboken, New Jersey, 2016
